# Sodium tanshinone IIA sulfonate protects against Aβ‐induced cell toxicity through regulating Aβ process

**DOI:** 10.1111/jcmm.15006

**Published:** 2020-01-27

**Authors:** Da‐Peng Zhang, Xin‐Yi Lu, Si‐Chen He, Wan‐Yan Li, Ran Ao, Feona Chung‐Yin Leung, Zhi‐Min Zhang, Qu‐Bo Chen, Shi‐Jie Zhang

**Affiliations:** ^1^ The First Affiliated Hospital of Guangzhou Medical University Guangzhou China; ^2^ Biological Resource Center The Second Affiliated Hospital of Guangzhou University of Chinese Medicine Guangzhou China; ^3^ Department of Neurology The People's Hospital of Baiyun District Guangzhou Guangzhou China; ^4^ LKS Faculty of Medicine School of Chinese Medicine The University of Hong Kong Pokfulam Hong Kong China; ^5^ Department of Neurology The Second Affiliated Hospital of Guangzhou University of Chinese Medicine Guangzhou China

**Keywords:** Aβ, Aβ degration, Aβ generation, sodium tanshinone IIA sulfonate

## Abstract

Sodium tanshinone IIA sulfonate (STS) has been reported to prevent Alzheimer's disease (AD). However, the mechanism is still unknown. In this study, two in vitro models, Aβ‐treated SH‐SY5Y cells and SH‐SY5Y human neuroblastoma cells transfected with APPsw (SH‐SY5Y‐APPsw cells), were employed to investigate the neuroprotective of STS. The results revealed that pretreatment with STS (1, 10 and 100 µmol/L) for 24 hours could protect against Aβ (10 µmol/L)‐induced cell toxicity in a dose‐dependent manner in the SH‐SY5Y cells. Sodium tanshinone IIA sulfonate decreased the concentrations of reactive oxygen species, malondialdehyde, NO and iNOS, while increased the activities of superoxide dismutase and glutathione peroxidase in the SH‐SY5Y cells. Sodium tanshinone IIA sulfonate decreased the levels of inflammatory factors (IL‐1β, IL‐6 and TNF‐α) in the SH‐SY5Y cells. In addition, Western blot results revealed that the expressions of neprilysin and insulin‐degrading enzyme were up‐regulated in the SH‐SY5Y cells after STS treatment. Furthermore, ELISA and Western blot results showed that STS could decrease the levels of Aβ. ELISA and qPCR results indicated that STS could increase α‐secretase (ADAM10) activity and decrease β‐secretase (BACE1) activity. In conclusion, STS could protect against Aβ‐induced cell damage by modulating Aβ degration and generation. Sodium tanshinone IIA sulfonate could be a promising candidate for AD treatment.

## 1 INTRODUCTION

Alzheimer's disease (AD) is increasingly severe in this century.^1^, ^2^ The clinical characteristics of AD are deficiencies in cognition.^3^ AD pathology is mainly characterized by excessive accumulation of toxic forms of amyloid‐β (Aβ), abnormally hyperphosphorylated tau and neurofibrillary tangles.^4^, ^5^ Some evidence suggests that overproduction of or reduced clearance of Aβ is occurred in the process of AD, which leading to the formation of Aβ plaques.^6^, ^7^ Aβ accumulation can induce deterioration of neurons and lower expressions of nerve growth factors, which lead to cognitive impairment and dementia.^8^, ^9^ Treatments that target Aβ process, decreasing the generation of Aβ or accelerating the clearance of Aβ, are considered to slow the progression of AD. However, up to now, all of the clinical trials targeting Aβ for AD treatment were reported failed, such as solanezumab, a monoclonal antibody targeting Aβ peptide.^10^ Thus, finding new therapeutic drugs is urgent.

Natural products are large potential sources of compounds for AD treatment.^11^ Sodium tanshinone IIA sulfonate (STS) is a derivative of Tanshinone IIA, which extracted from the dried roots of Danshen (*Salvia miltiorrhiza*). A large number of studies have shown that STS could protect against cardiovascular diseases.^12^, ^13^, ^14^, ^15^ Besides the well‐known cardioprotective effect, STS possesses neuroprotective activity against neural dysfunction.^16^, ^17^, ^18^ Previous studies suggested that STS have some pharmacological actions, such as anti‐oxidative stress,^19^, ^20^ anti‐inflammation.^13^, ^21^ However, STS has not yet been reported to have any Aβ‐regulation effect. Considering during AD process, Aβ aggregation can damage and cause neuronal death by inducing oxidative stress and neuroinflammation.^22^, ^23^ Therefore, it was hypothesized that STS could display the neuroprotective effects through modulating Aβ process.

In this study, two in vitro models, Aβ‐treated SH‐SY5Y cells and SH‐SY5Y human neuroblastoma cells transfected with APPsw (SH‐SY5Y‐APPsw cells), were employed to investigate the neuroprotective of STS. Different doses (1, 10 or 100 µmol/L) of STS were used to treat cells. We revealed that STS could obviously protect against Aβ‐induced cell toxicity through modulating Aβ degration and generation.

## 2 MATERIALS AND METHODS

### 2.1 Cell culture

**Figure 1:**
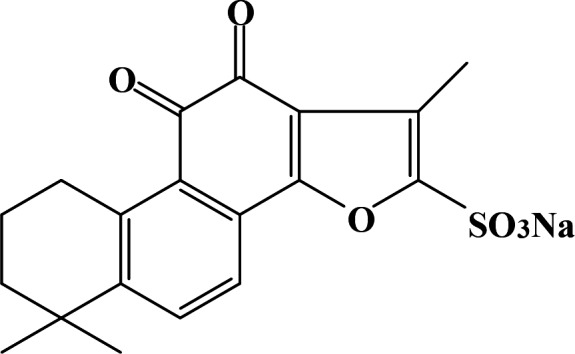
Molecular structure of STS

Both SH‐SY5Y cells and SH‐SY5Y‐APPsw cells were cultured in DMEM medium with 10% foetal bovine serum (Thermo Fisher Scientific), 1× antibiotic (Thermo Fisher Scientific) was added into the medium. The cells were cultured in a humidified incubator under standard conditions of 37°C with 4%‐5% CO_2_. The SH‐SY5Y cells were treated with 1, 10 or 100 µmol/L Sodium tanshinone IIA sulfonate (STS, MedChem Express, Figure 1) for 24 hours and then treated with 10 µmol/L Aβ (Sigma‐Aldrich) for 24 hours. The SH‐SY5Y‐APPsw cells were treated with 1, 10 or 100 µmol/L STS for 24 hours.

### 2.2 Cell viability

The SH‐SY5Y cells were seeded in the 96‐well plates. The cell viability was measured by MTT assay.

### 2.3 Reactive oxygen species (ROS) level

The cells were collected and centrifuged. The 2′7′‐dichlorofluorescein diacetate (DCFH‐DA) fluorescent dye method (Invitrogen) was used to measure the ROS level. The presence of ROS can convert non‐fluorescent DCFH‐DA to fluorescent dichlorofluorescein (DCF).

### 2.4 Malondialdehyde (MDA) level

The cells were collected to detect the level of MDA by using the kit (Nianjing Jiancheng Bioengineering Institute) according to the manufacturer's instructions.

### 2.5 Superoxide dismutase (SOD) and glutathione peroxidase (GSH‐Px) activities

The cells were collected to detect the activities of SOD and GSH‐Px by using the kits (Nianjing Jiancheng Bioengineering Institute) according to the manufacturer's instructions.

### 2.6 Nitric oxide (NO) level

The cells were collected to detect the level of NO by using the kit (Abcam) according to the manufacturer's instructions.

### 2.7 ELASA

The SY5Y cells were collected. The concentrations of IL‐1β, IL‐6, TNF‐α and iNOS were measured by using the ELISA kits (Thermo Fisher Scientific) according to the manufacturer's instructions.

The SH‐SY5Y‐APPsw cells were collected. The levels of Aβ1‐40 and Aβ1‐42 were measured by using the ELISA kits (Invitrogen) according to the manufacturer's instructions. The activities of α‐, β‐ and γ‐secretase were measured by using the ELISA kits (R&D Systems) according to the manufacturer's instructions.

### 2.8 Western blot analysis

The cells were collected and lysed in RIPA buffer. The lysate was collected and extracted the protein. The protein was separated by SDS‐PAGE gel and then migrated to PVDF membranes. The membranes were incubated with anti‐neprilysin (NEP), anti‐insulin‐degrading enzyme (IDE), anti‐Aβ and anti‐β‐actin primary antibodies and then incubated with horseradish peroxidase‐conjugated anti‐rabbit secondary antibody. Finally, the exposures of membranes were analysed.

### 2.9 Quantitative PCR

The cells were collected, and the total RNA was isolated by using the RNeasy kit (Hilden) and reverse‐transcribed using SuperScript III Reverse Transcriptase (Invitrogen). Forward and reverse primers were as follows: ADAM metallopeptidase domain 10 (ADAM10): For, 5′‐TTCTCCCTCCGGATCGATGT‐3′, Rev, 5′‐ATACTGACCTCCCATCCCCG‐3′; beta‐secretase 1 (BACE1): For, 5′‐ACTTTACACTCTGTTCTGGGTGG‐3′, Rev, 5′‐ACCACAAAGCCTGGCAATCTC‐3′; presenilin 1 (PSEN1): For, 5′‐ AATGACGACAACGGTGAGGG‐3′, Rev, 5′‐CCAGATTAGGTGCTTCCCCG‐3′; β‐actin: For, 5′‐AGAGCTACGAGCTGCCTGAC‐3′, Rev, 5′‐AGCACTGTGTTGGCGTACAG‐3′.

### 2.10 Statistical analysis

The data were analysed by using Student's *t* test and ANOVA by SPSS 19.0 statistical software (IBM). The results were expressed as the mean ± SEM. The differences were considered as statistically significant at *P* < .05.

## 3 RESULTS

### 3.1 STS ameliorates Aβ‐induced cell toxicity in SH‐SY5Y cells

**Figure 2:**
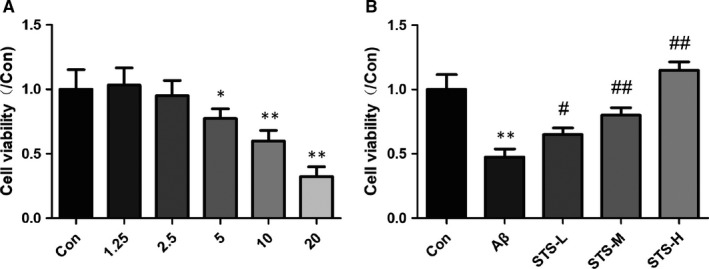
STS protects against Aβ‐induced cell toxicity in SH‐SY5Y cells. (A) MTS cell viability assay was performed in SH‐SY5Y cells after exposure to different concentrations of Aβ (1.25, 2.5, 5, 10 and 20 µmol/L) for 24 h. (B) MTS cell viability of Aβ (10 µmol/L)‐treated SY5Y cells after STS preprotection. STS‐L: 1 µmol/L, STS‐M: 10 µmol/L, STS‐H: 100 µmol/L. Experimental values were expressed as mean ± SEM. *P < .05, **P < .01 vs Con group; ^#^ *P* < .05, ^##^ *P* < .01 vs Aβ group

In order to prove whether STS had the neuroprotective effect, the Aβ‐treated SH‐SY5Y cell model was employed. We firstly screened the best concentration of Aβ. Different dosages of Aβ (1.25, 2.5, 5, 10 and 20 µmol/L) were added into the cultured medium for 24 hours. As revealed in the MTT test (Figure 2A), the concentrations of Aβ (5, 10 and 20 µmol/L) treatment caused cell injury obviously. The concentration of Aβ (10 µmol/L) was around the median lethal dose. Thus, 10 µmol/L Aβ was selected for the further study. We next studied the neuroprotective effect of STS on Aβ‐treated SH‐SY5Y cells. The SH‐SY5Y cells were pretreated with different concentrations of STS (1, 10 and 100 µmol/L) for 24 hours, and then treated with 10 µmol/L Aβ for 24 hours. Result indicated that STS could prevent against Aβ‐induced cell toxicity in a dose‐dependent manner (Figure 2B). These data indicated that STS had neuroprotective effect against Aβ‐induced cell toxicity.

### 3.2 STS ameliorates oxidative stress, nitrosative stress and neuroinflammation in Aβ‐treated SH‐SY5Y cells

**Figure 3:**
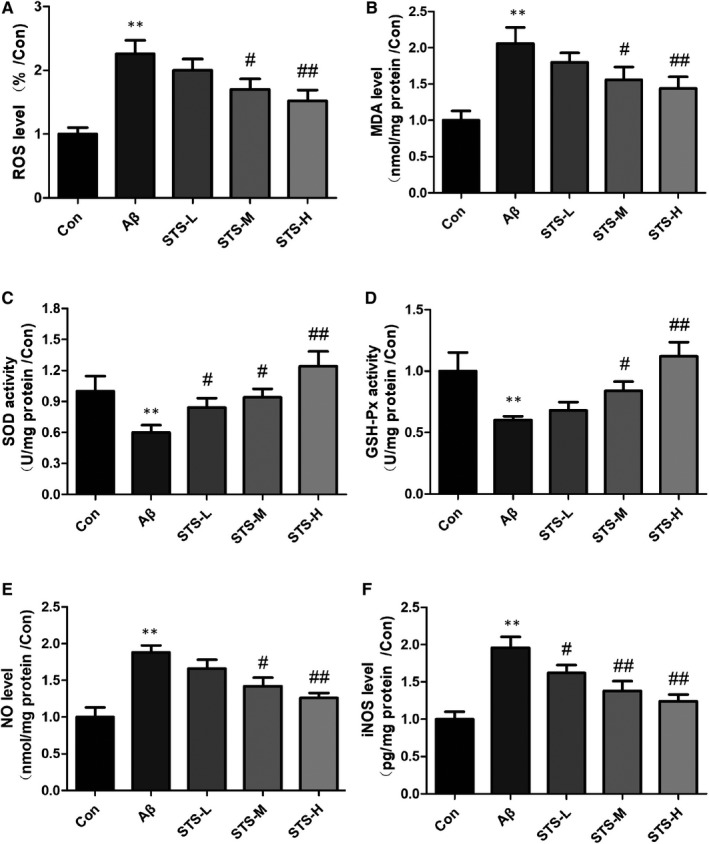
STS ameliorates oxidative and nitrosative stress in Aβ‐treated SH‐SY5Y cells. The levels of ROS (A), MDA (B), the activities of SOD (C), GSH‐Px (D) and the levels of NO (E), iNOS (F) in SH‐SY5Y cells. STS‐L: 1 µmol/L, STS‐M: 10 µmol/L, STS‐H: 100 µmol/L. Experimental values were expressed as mean ± SEM. *P < .05, **P < .01 vs Con group; ^#^ *P* < .05, ^##^ *P* < .01 vs Aβ group

**Figure 4:**
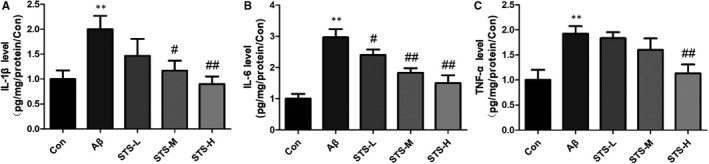
STS reverses neuroinflammation in Aβ‐treated SH‐SY5Y cells. The levels of IL‐1β (A), IL‐6 (B) and TNF‐α (C) were detected by ELISA in SH‐SY5Y cells. STS‐L: 1 µmol/L, STS‐M: 10 µmol/L, STS‐H: 100 µmol/L. Experimental values were expressed as mean ± SEM. *P < .05, **P < .01 vs Con group; ^#^ *P* < .05, ^##^ *P* < .01 vs Aβ group

Oxidative and nitrosative stress statements are observed in the AD patients' brain.^24^, ^25^ As shown in Figure 3, oxidative stress (increased levels of ROS and MDA, decreased activities of SOD and GSH‐Px) and nitrosative stress (increased levels of NO and iNOS) were observed in Aβ‐treated SH‐SY5Y cells. While, STS pretreatment decreased the levels of ROS, MDA, NO and iNOS, and increased the activities of SOD and GSH‐Px significantly. In addition, Aβ accumulation can also induce neuroinflammation in the AD patients' brain.^26^, ^27^ In this study, Aβ‐treatment increased the neuroinflammatory factors (IL‐1β, IL‐6 and TNF‐α) in SH‐SY5Y cells. Sodium tanshinone IIA sulfonate pretreatment significantly decreased these neuroinflammatory factors (Figure 4). These findings suggested that the neuroprotective effects of STS might be in connection with its anti‐oxidative and nitrosative stress and anti‐inflammatory abilities.

### 3.3 STS improves the expressions of Aβ‐degrading enzymes in Aβ‐treated SH‐SY5Y cells

**Figure 5:**
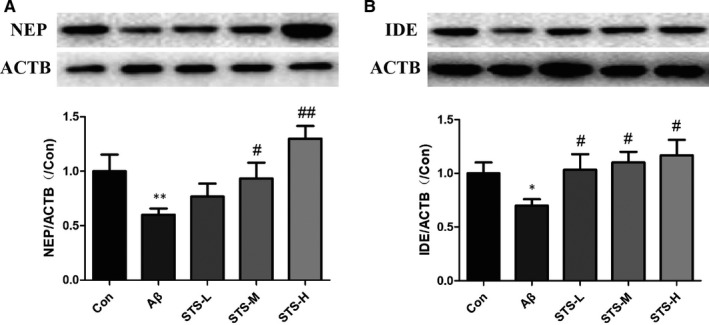
STS improves the protein expressions of Aβ degrading enzymes in Aβ‐treated SH‐SY5Y cells. NEP (A) and IDE (B) protein levels were detected by Western blotting in SH‐SY5Y cells. STS‐L: 1 µmol/L, STS‐M: 10 µmol/L, STS‐H: 100 µmol/L. Experimental values were expressed as mean ± SEM. *P < .05, **P < .01 vs Con group; ^#^ *P* < .05, ^##^ *P* < .01 vs Aβ group

NEP and IDE are two important Aβ‐degrading enzymes in the cell.^28^, ^29^ As shown in Figure 5, Aβ‐treatment caused the cell damage and decreased the protein expressions of NEP and IDE in SH‐SY5Y cells. In contrast, STS pretreatment prevented against the decreases of NEP and IDE. These data indicated that STS could protect against Aβ‐induced cell toxicity by modulating Aβ degration.

### 3.4 STS inhibits Aβ generation in SH‐SY5Y‐APPsw cells

**Figure 6:**
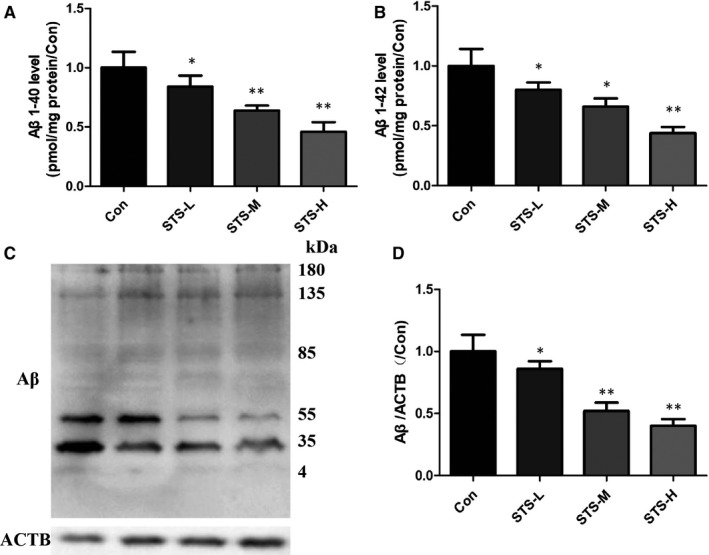
STS decreases Aβ level in SH‐SY5Y‐APPsw cells. Aβ1‐40 (A) and Aβ1‐42 (B) levels were measured by ELISA in SH‐SY5Y‐APPsw cells. Aβ protein level was detected by Western blotting (C and D) in SH‐SY5Y‐APPsw cells. STS‐L: 1 µmol/L, STS‐M: 10 µmol/L, STS‐H: 100 µmol/L. Experimental values were expressed as mean ± SEM. *P < .05, **P < .01 vs Con group

**Figure 7:**
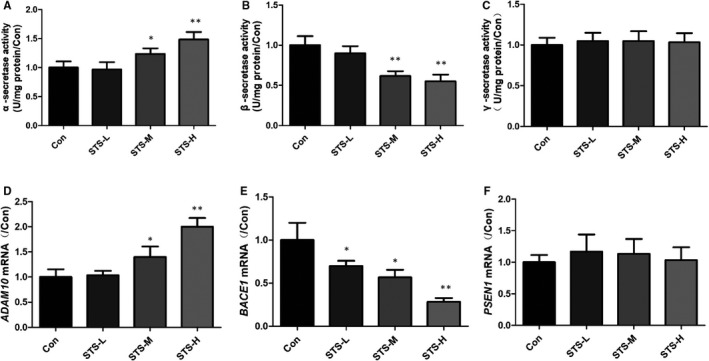
STS affects Aβ generating enzymes in SH‐SY5Y‐APPsw cells. α‐ (A), β‐ (B) and γ‐secretase (C) activities were measured by ELISA in SH‐SY5Y‐APPsw cells. The mRNA expressions of ADAM 10 (D); BACE1 (E) and PS1 (F) were determined by qPCR in SH‐SY5Y‐APPsw cells. Experimental values were expressed as mean ± SEM. *P < .05, **P < .01 vs Con group

Furthermore, we also studied the effect of STS on Aβ generation. A SH‐SY5Y cell line overexpressing the human APP Swedish mutant (SH‐SY5Y‐APPsw) was used for investigation. The levels of Aβ1‐40 and Aβ1‐42 were significantly decreased after STS treatment in a dose‐dependent manner (Figure 6A,B). Western blot result also showed the same effect (Figure 6C,D). We next studied the effect of STS on APP cleavage process. Amyloid precursor protein is mainly cleaved by three enzymes, α‐, β‐ and γ‐secretases. ELISA results showed that STS increased α‐secretase activity and decreased β‐secretase activity, while did not affect the γ‐secretase activity (Figure 7A‐C). Inconsistently, qPCR results indicated that STS increased ADAM10 and decreased BACE1 mRNA expression. PS1 mRNA expression was not changed (Figure 7D‐F). Thus, it was revealed that STS could protect against Aβ‐induced cell toxicity by inhibiting Aβ generation.

## 4 DISCUSSION

In this study, we studied the neuroprotective effect of STS against Aβ process in two in vitro models, Aβ‐treated SH‐SY5Y cells and SH‐SY5Y‐APPsw cells. Pretreatment of STS (1, 10 and 100 µmol/L) could relieve Aβ‐induced cell impairment. In additionally, STS could protect cells against Aβ‐induced oxidative stress and inflammation. Mechanism studies showed that the neuroprotective effect of STS might be through modulating Aβ degration and generation.

The impairment of cognitive function is the characteristic of AD. The pathological product, Aβ (the main component of amyloid plaques), acts as a central role in AD process.^30^, ^31^, ^32^, ^33^ Aβ can aggregate to form oligomers. The Aβ oligomers are the most toxic to neuron.^34^ In this study, Aβ was treated to SH‐SY5Y cells to induce cytotoxicity. MTT results revealed that the STS treatment ameliorated Aβ‐induce cytotoxicity. Aβ accumulation can exacerbate oxidative stress and inflammation, which damage proteins, DNA, lipids and other compounds.^35^ Oxidative, nitrosative stress and inflammation are playing crucial roles in cell homeostasis and apoptosis. Oxidative, nitrosative stress and inflammation in neuron can cause dysfunction of neural junction, and then induce the nervous system damage.^36^ The antioxidant enzymes were significantly decreased in AD mice.^37^, ^38^ In this study, the primary indicators of oxidative and nitrosative stress were obviously increased after Aβ treatment. The activities of the antioxidant enzymes were decreased. Sodium tanshinone IIA sulfonate pretreatment reversed the Aβ‐induced oxidative and nitrosative stress. Inflammation also plays an important role in AD. Some inflammatory factors, such as IL‐1β, IL‐6 and TNF‐α, were observed in AD brain.^39^, ^40^, ^41^ In our study, excessive IL‐1β, IL‐6 and TNF‐α were produced in the Aβ‐treated SH‐SY5Y cells. Sodium tanshinone IIA sulfonate suppressed the production of these inflammatory factors. These findings suggested that the anti‐Aβ cytotoxicity effect of STS could be related to the anti‐oxidative stress and anti‐inflammation capacity.

Amyloid precursor protein (APP) is an integral membrane protein, which can be cleaved by α‐ (the extracellular region), β‐ (the extracellular region) and γ‐secretase enzymes. Aβ is the cleaved product of APP by β‐ and γ‐secretase under pathological conditions. Amyloid precursor protein is mainly cleaved by α‐secretase and γ‐secretase under normal physiological conditions.^42^ The cleavage by α‐secretase can prevent the generation of Aβ. Thus, increasing ADAM10 (α‐secretase), or inhibiting of BACE1 (β‐secretase), can avoid Aβ generation. In our study, STS could decrease the levels of Aβ1‐40 and Aβ1‐42 in SH‐SY5Y‐APPsw cells. Both ELISA and qPCR results found that STS increased the activity of α‐secretase and decreased the activity of β‐secretase. However, STS did not affect the activity of γ‐secretase. In addition, Aβ clearance is another important pathway to protect against AD process. Aβ can be cleared through the intracellular pathway or the extracellular pathway. Blood‐brain barrier (BBB) transport redominates the extracellular pathway.^43^ Intracellular pathway is mainly occurred in neurons or glia.^44^ The enzymatic breakdown‐induced degradation clearance is the major pathway, including NEP and IDE.^45^, ^46^, ^47^ In this study, STS increased the protein expressions of NEP and IDE in SH‐SY5Y cells. The above results demonstrated that STS could protect against Aβ‐induced cell toxicity through modulating Aβ degration and generation.

In conclusion, the study provides some evidence that STS can alleviate Aβ‐induced cell toxicity by inhibiting oxidative stress and neuroinflammation. This neuroprotective effect of STS might be through regulating Aβ degration and generation. Sodium tanshinone IIA sulfonate might be developed as a new anti‐AD drug. However, whether STS could affect Aβ transport is still unknown. Further studies are needed.

## CONFLICT OF INTEREST

The authors declare that they have no conflicts of interest.

## AUTHORS' CONTRIBUTIONS

Da‐Peng Zhang and Xin‐Yi Lu finished most of the experiments; Si‐Chen He, Wan‐Yan Li, Ran Ao and Zhi‐Min Zhang helped data organization. Qu‐Bo Chen confirmed the data. Shi‐Jie Zhang designed the experiments and modified manuscript.

## Data Availability

The data used to support the findings of this study are available from the corresponding author upon request.
